# A cost-effective proof-of-concept *ex vivo* perfusion model for myocardial ischemia–reperfusion injury in wistar rats: enhancing accessibility in resource-constrained settings

**DOI:** 10.3389/fcvm.2026.1820520

**Published:** 2026-04-29

**Authors:** Sainath P, Shakta Mani Satyam, Abdul Rehman, Sanjay Bharati, Prakashchandra Shetty, Mohamed El-Tanani, Akheruz Zaman Ahmed, Rashmi Kumari, Muaweya Sufian Albadawi, Firas Assaf, Seyed Mohammad Seyed Morteza Hashemi, Faisal Aslam Khan, Mohmed Krem Adnan Doukarli, Marwan Mohamed Hassan Rustom Robari, Murk Jai Pal Paryani, Varun Kumar Singh, Ganesh Kamath S

**Affiliations:** 1 Department of Perfusion Technology, Manipal College of Health Professions, Manipal Academy of Higher Education, Manipal, India; 2 Department of Pharmacology, RAK College of Medical Sciences, RAK Medical and Health Science University, Ras al Khaimah, United Arab Emirates; 3 Department of Pathological Sciences, College of Medicine, Ajman University, Ajman, United Arab Emirates; 4 Department of Nuclear Medicine, Manipal College of Health Professions, Manipal Academy of Higher Education, Manipal, India; 5 Department of Anatomy, Manipal University College Malaysia, Melaka, Malaysia; 6 RAK College of Pharmacy, RAK Medical and Health Science University, Ras al Khaimah, United Arab Emirates; 7 Department of Anatomy, Fakhruddin Ali Ahmed Medical College, Srimanta Sankaradeva University of Health Sciences, Assam, India; 8 A. J. Institute of Hospital Management, Mangalore, India; 9 National Reference Lab, Redcliffe Labs, Noida, Uttar Pradesh, India; 10 Department of Cardiovascular and Thoracic Surgery, Kasturba Medical College, Manipal Academy of Higher Education, Manipal, India

**Keywords:** cardioprotective research, ex-vivo heart perfusion, ischemic heart disease model, langendorff model, myocardial ischemia-reperfusion injury, preclinical cardiovascular model, TTC staining

## Abstract

**Introduction:**

Myocardial ischemia–reperfusion injury (IRI) continues to impede the advancement of effective cardioprotective therapies. Experimental limitations, particularly the lack of accessible and cost-effective *ex vivo* platforms, restrict mechanistic and translational research. This study aimed to develop a simplified, resource-conscious proof-of-concept *ex vivo* rat heart perfusion model capable of inducing key features of myocardial IRI, rather than establishing a fully validated replacement for conventional Langendorff systems.

**Methods:**

Twelve adult male Wistar rats were randomly assigned to a non-ischemic control group (*n* = 6) or an IRI group (*n* = 6). Following a 10 min stabilization period, hearts in the IRI group underwent 30 min of global ischemia using potassium-based Krebs–Henseleit buffer (KHB) cardioplegic solution, followed by 60 min of reperfusion. Control hearts received continuous perfusion without ischemic insult. Coronary flow was assessed to evaluate functional changes. Myocardial injury was quantified using triphenyl tetrazolium chloride (TTC) staining, and structural alterations were examined through histopathological analysis.

**Results:**

The IRI group demonstrated an approximately 4.7-fold reduction in coronary flow during early reperfusion, reflecting acute vascular dysfunction. TTC staining revealed significant infarct development compared to controls. Histopathological examination confirmed cardiomyocyte degeneration, cytoplasmic vacuolization, inflammatory cell infiltration, and interstitial edema. Functional impairment strongly correlated with structural myocardial injury, demonstrating consistent induction of myocardial injury within the experimental setting.

**Discussion:**

This simplified *ex vivo* model reproduces fundamental features of myocardial IRI within a resource-conscious framework. However, it should be interpreted as a proof-of-concept platform rather than a replacement for conventional Langendorff systems.

## Graphical Abstract

**Figure:**
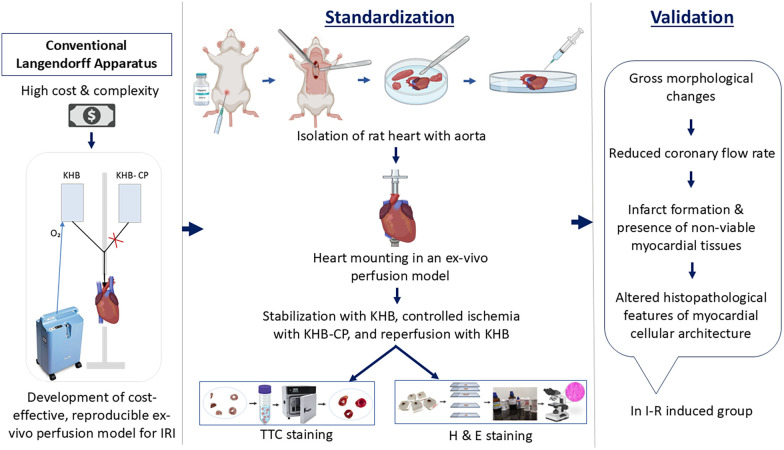

## 1 Introduction

Cardiovascular diseases (CVDs) continue to be the foremost cause of morbidity and mortality globally, with myocardial ischemia–reperfusion injury (IRI) representing a critical determinant of adverse cardiac outcomes during clinical interventions such as primary percutaneous coronary intervention and coronary artery bypass grafting (1, 2). Despite significant advances in therapeutic strategies, IRI remains a major unsolved clinical problem, as reperfusion although essential to restore myocardial blood flow paradoxically exacerbates cardiac injury through mechanisms such as oxidative stress, calcium overload, and inflammation (3). Understanding these processes in depth is vital for the development of effective therapeutic interventions.

Given the multifaceted complexity of human cardiovascular physiology, direct study of IRI in clinical settings is challenging. Animal models have therefore become indispensable for deciphering the underlying molecular and cellular mechanisms as well as for preclinical evaluation of selected drugs. Among these models, the isolated heart perfusion technique particularly the Langendorff model first described in 1895 has emerged as a cornerstone in experimental cardiovascular research (4–8).

The Langendorff preparation offers a unique advantage by allowing the assessment of intrinsic cardiac function independent of systemic influences such as neurohormonal regulation, circulating factors, or interactions with other organs. Through retrograde perfusion of the aorta with a physiologically formulated buffer solution, the method provides oxygen and nutrients directly to the coronary circulation, enabling the isolated heart to retain its contractile activity in a manner analogous to *in vivo* physiology (9). This highly controlled environment not only facilitates the evaluation of both physiological and pharmacological responses but also allows for precise induction of ischemia and reperfusion, thereby creating a reproducible platform to investigate myocardial IRI (4, 10, 11).

Over decades, studies employing the Langendorff apparatus have been instrumental in elucidating the multifactorial mechanisms of IRI, implicating oxidative stress, calcium dysregulation, mitochondrial dysfunction, and inflammasome-mediated inflammation as central contributors (12–14). These mechanistic findings closely mirror pathophysiological events in human IRI, particularly oxidative and inflammatory cascades, thus reinforcing the translational value of this model. Importantly, experimental interventions targeting these pathways within the Langendorff system have successfully attenuated myocardial damage, demonstrating its utility for therapeutic discovery with direct clinical implications (15–18).

However, despite its wide utility, the Langendorff preparation presents significant technical and logistical challenges. Beyond the perfusion system itself, successful experimentation depends on standardized execution of critical steps including heart excision, mounting of the organ, stabilization, and precise ischemia–reperfusion protocols. Variability in these steps can lead to inconsistent outcomes, compromised myocardial viability, and poor reproducibility. Moreover, the requirement of specialized apparatus, precise pressure control, and advanced laboratory infrastructure renders this model resource intensive. For laboratories in resource-constrained environments, these challenges pose a substantial barrier to adopting the Langendorff system, thereby limiting opportunities for cardiovascular research and training.

This presents a clear research problem- although the Langendorff model remains the gold standard for *ex vivo* studies of myocardial IRI, its technical demands and high costs hinder widespread adoption. Consequently, there is an unmet need for simplified, standardized, and cost-effective protocols that preserve the scientific rigor and reproducibility of the model while lowering the barrier to entry for laboratories with limited infrastructure. Therefore, the aim of this study was to develop and standardize a resource-conscious proof-of-concept *ex vivo* perfusion model of myocardial IRI in Wistar rats, with an emphasis on procedural reproducibility and accessibility for resource-constrained laboratories.

In this context, the present study sought to develop and standardize a resource-conscious proof-of-concept *ex vivo* perfusion model of myocardial IRI in Wistar rats using a cost-effective alternative to the conventional Langendorff apparatus. Wistar rats were chosen as they are well-characterized in cardiovascular research, offering physiological reproducibility and translational relevance for modeling IRI. Preclinical studies in Wistar rats have demonstrated the utility of this species for investigating diverse pathophysiological processes and therapeutic interventions, ranging from hepatoprotection and metabolic modulation to wound healing and oxidative stress mitigation (19–26). Importantly, our approach focused not only on creating a robust perfusion system but also on streamlining critical technical steps- heart excision, mounting, stabilization, and reperfusion protocols so that the model could be replicated consistently in diverse research settings.

The novelty of this work lies in bridging the gap between high-resource laboratories and resource-constrained environments by providing an accessible and simplified experimental platform that retains the essential physiological and pathophysiological features of IRI. By addressing both technical and economic barriers, this study provides a preliminary framework for accessible experimentation that may support mechanistic exploration in resource-limited settings, while requiring further validation for broader application.

## 2 Materials and methods

### 2.1 Drugs, chemicals and labware

General anesthetic agents, Ketamine Hydrochloride Injection I.P. (Aneket®, 250 mg/5 mL), Xylazine Injection U.S.P. (XYLAXIN®, 30 mL), and Heparin Injection I.P. (HEPAGLAN®-5, 5,000 IU/5 mL) and Philips Everflo Oxygen Concentrator were procured from Radha Medicals, Manipal, India. Chemicals/Reagents required for the preparation of Krebs-Henseleit Buffer (KHB), KHB-based cardioplegic (KHB-CP) solutions and histopathological analysis were procured from Sigma Aldrich-Merck, Bangalore, India. Tetrazolium Salt 99% AR was obtained from Loba Chemie Pvt. Ltd. Maharashtra, India. All reagents and chemicals used were of analytical grade.

Medical-grade ¼-inch polyvinyl chloride (PVC) tubing, ¼-inch polycarbonate Y-connectors with luer lock, cardioplegia connector, plastic spikes and stoppers, surgical scalpel blade no. 22, blood set regulators for flow control, metal clamps to occlude perfusate flow, and 20-gauge needles for aortic cannulation were obtained from Sri Mahalasa Traders, Udupi, India.

### 2.2 Animals

A total of 12 adult male Wistar rats (150–200 g; 10–12 weeks old) were procured from the Central Animal House Facility, Manipal Academy of Higher Education (MAHE), Manipal, India, an approved animal breeding centre. The animals were maintained under a 12 h light/dark cycle, with a relative humidity of 40%–60% and a constant ambient temperature of 22–24 °C (19, 21, 24). The animals were provided with a standard rat pellet diet obtained from a local supplier and had unlimited access to tap water. A one-week acclimatization period was allowed prior to the initiation of experimental procedures to ensure adaptation to the laboratory environment (19, 20, 27). This study was conducted in full compliance with the 3Rs principles- Replacement, Reduction, and Refinement- to ensure humane care and welfare of the animals. All experimental procedures were conducted in accordance with the ethical guidelines established by the Institutional Animal Ethics Committee (IAEC), with prior approval obtained (IAEC/KMC/74/2021). The study also conforms to the standards set by the Committee for the Control and Supervision of Experiments on Animals (CCSEA), Government of India. Furthermore, the study followed the ARRIVE (Animal Research: Reporting of *in vivo* Experiments) guidelines to promote transparency, reproducibility, and ethical integrity in animal research, emphasizing the minimization of animal distress and the maximization of scientific value. Male Wistar rats were chosen to avoid hormonal fluctuations associated with estrous cycles in females, which may influence myocardial susceptibility to ischemia–reperfusion injury and confound results. This standardization enhances reproducibility in a proof-of-concept study. The sample size (*n* = 12; 6 control and 6 IRI) was determined using pilot-effect estimates and power justification based on published ex-vivo myocardial IRI datasets. Prior studies and our preliminary observations demonstrated large, expected effect sizes (Cohen's d ≈ 1.3) for TTC-derived infarct percentage and histopathological injury. Using a two-sample *t*-test (*α* = 0.05, power = 0.80), a minimum of six animals per group was required to detect biologically meaningful differences (28). This sample size balances statistical rigor with ethical stewardship under the 3Rs and CCSEA guidelines.

### 2.3 Assembly of extracorporeal circuit and Ex-vivo heart perfusion model

**Figure 1:**
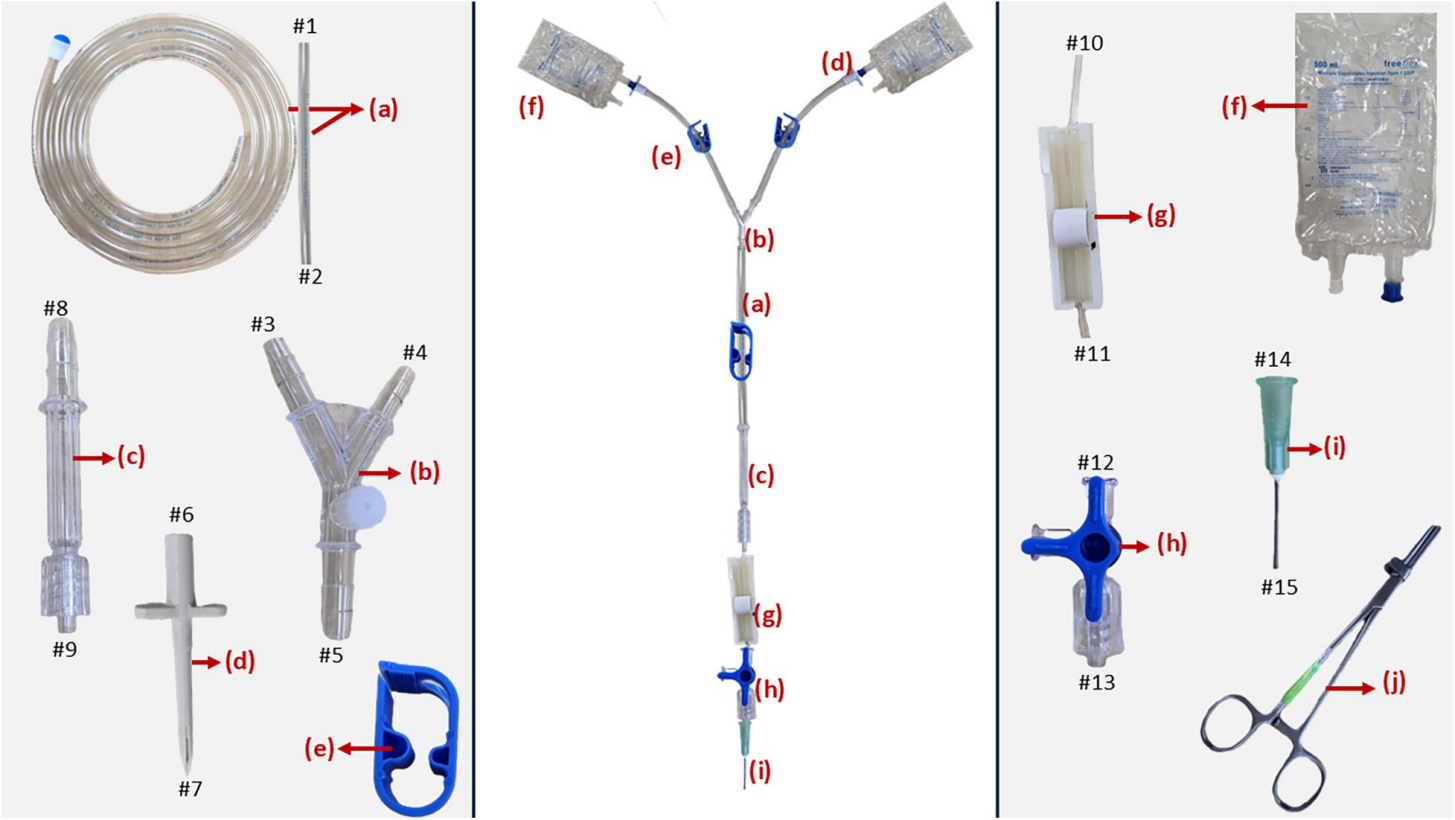
Various components of extracorporeal circuit and the fully assembled circuit. **(A)** ¼-inch polyvinyl chloride tubing, **(B)** ¼-inch polycarbonate Y-connector with luer lock, **(C)** cardioplegia connector, **(D)** plastic spikes, **(E)** plastic stopper, **(F)** empty Plasma-Lyte fluid bag, **(G)** IV set flow regulator, **(H)** three-way valve, **(I)** 20-gauge blunt needle, **(J)** metal clamp.

For assembly of the extracorporeal circuit, all required disposables were obtained prior to assembly. Two equal lengths of ¼-inch PVC tubing were cut using a surgical scalpel blade (no. 22), and their proximal ends (#1) were connected to the two arms of a polycarbonate Y-connector (#3 and #4). The distal ends of these tubing (#2) were attached to plastic spikes (#6), which were inserted into repurposed Plasma-Lyte-A bags (#7) containing Krebs–Henseleit buffer (KHB) and KHB-based cardioplegic (KHB-CP) solutions, respectively. The luer-lock arm of the Y-connector was sealed with a non-vented luer cap, which was later used for circuit de-airing. A third length of ¼-inch PVC tubing was connected from the third arm of the Y-connector (#5) to a cardioplegia connector (#8). An IV set flow regulator line (#10) was then attached to the outlet of the cardioplegia connector (#9) to permit precise control of flow. Plastic stoppers were placed on each of the ¼-inch PVC tubing to allow temporary occlusion as required. The terminal end of the IV set flow regulator line (#11) was connected to a three-way valve inlet (#12), with its outlet (#13) fitted to a 20-gauge blunt needle (#14), which functioned as the arterial cannula for myocardial perfusion (Figure 1).

For oxygenation, the outlet of an oxygen concentrator was connected via ¼-inch PVC tubing to the KHB reservoir bag, ensuring continuous delivery of oxygenated perfusate to the myocardium. Temperature regulation was maintained using a heater–cooler system. The KHB bag was placed within a rolled-up warming water blanket, through which water at 37 °C was circulated from the heater–cooler unit. This arrangement ensured that the perfusate temperature was consistently maintained at 35 ± 2 °C, thereby preserving physiological conditions throughout the procedure.

### 2.4 Preparation of KHB and KHB-CP solution

Physiological salt solutions- Krebs-Henseleit Buffer (KHB) and KHB-based cardioplegic (KHB-CP) solutions were freshly prepared on the day of each experiment to ensure optimal stability and functionality of all components. These solutions contain essential electrolytes and substrates that are critical for maintaining physiological ionic balance, myocardial contractility, and energy supply during *ex vivo* heart perfusion. Sodium chloride and potassium chloride regulate osmolarity and excitability, calcium and magnesium ions support contractile function and vascular tone, glucose serves as an energy source, and bicarbonate buffers pH. The physiological Krebs–Henseleit Buffer (KHB) was prepared with a pH at 7.35–7.40 and an osmolarity of 285–295 mOsm·kg⁻¹, providing a stable and physiologically relevant ionic environment for *ex vivo* perfusion. However, the KHB-based cardioplegic solution (KHB-CP) was specifically formulated with elevated potassium and magnesium concentrations and reduced calcium levels to induce rapid myocardial arrest and enhance tissue protection during isolation and perfusion procedures. The final KHB-CP preparation was formulated to a pH of 7.8 and an osmolarity of 380 mOsm·kg⁻¹, reflecting its modified ionic composition intended for cardioplegic efficacy (Table 1) (28–30).

**Table 1:** Composition of Krebs-Henseleit Buffer (KHB) solution and Krebs-Henseleit Buffer-based cardioplegic (KHB-CP) solution.

Component	Krebs-Henseleit buffer solution (g/L)	Krebs-Henseleit buffer based cardioplegic solution (g/L)
Sodium Chloride (NaCl)	6.95	8.36
Potassium Chloride (KCl)	0.35	1.86
Calcium Chloride (CaCl_2_.2H_2_O)	0.37	0.044
Magnesium Sulphate (MgSO_4_.7H_2_O)	0.30	3.94
Glucose	1.98	1.98
Sodium Bicarbonate (NaHCO_3_)	2.10	2.10
Potassium dihydrogen phosphate (KH_2_PO_4_)	0.16	0.163

### 2.5 Experimental design

A total of 12 male Wistar rats (150–200 g; 10-12 weeks old) were randomly assigned to two groups (*n* = 6 per group): Group I—Non-ischemic control and Group II—Ischemia-reperfusion (I-R) induced. In the I–R group, each heart first underwent a 10 min stabilization period to ensure hemodynamic equilibrium under baseline perfusion conditions. Following stabilization, global ischemia was induced by administering the KHB-cardioplegic (KHB-CP) solution for 30 min, after which reperfusion was initiated and maintained for 60 min using standard KHB solution.

In contrast, hearts in the non-ischemic control group also underwent an initial 10 min stabilization period but were not subjected to ischemia or reperfusion. Instead, they received a continuous, uninterrupted perfusion with physiological saline buffer (KHB) and atmospheric air supplied through an oxygen concentrator throughout the entire experimental duration. This group served as a baseline control to verify the intrinsic stability, functional integrity, and reliability of the ex-vivo perfusion system in the absence of ischemic insult.

**Figure 2:**
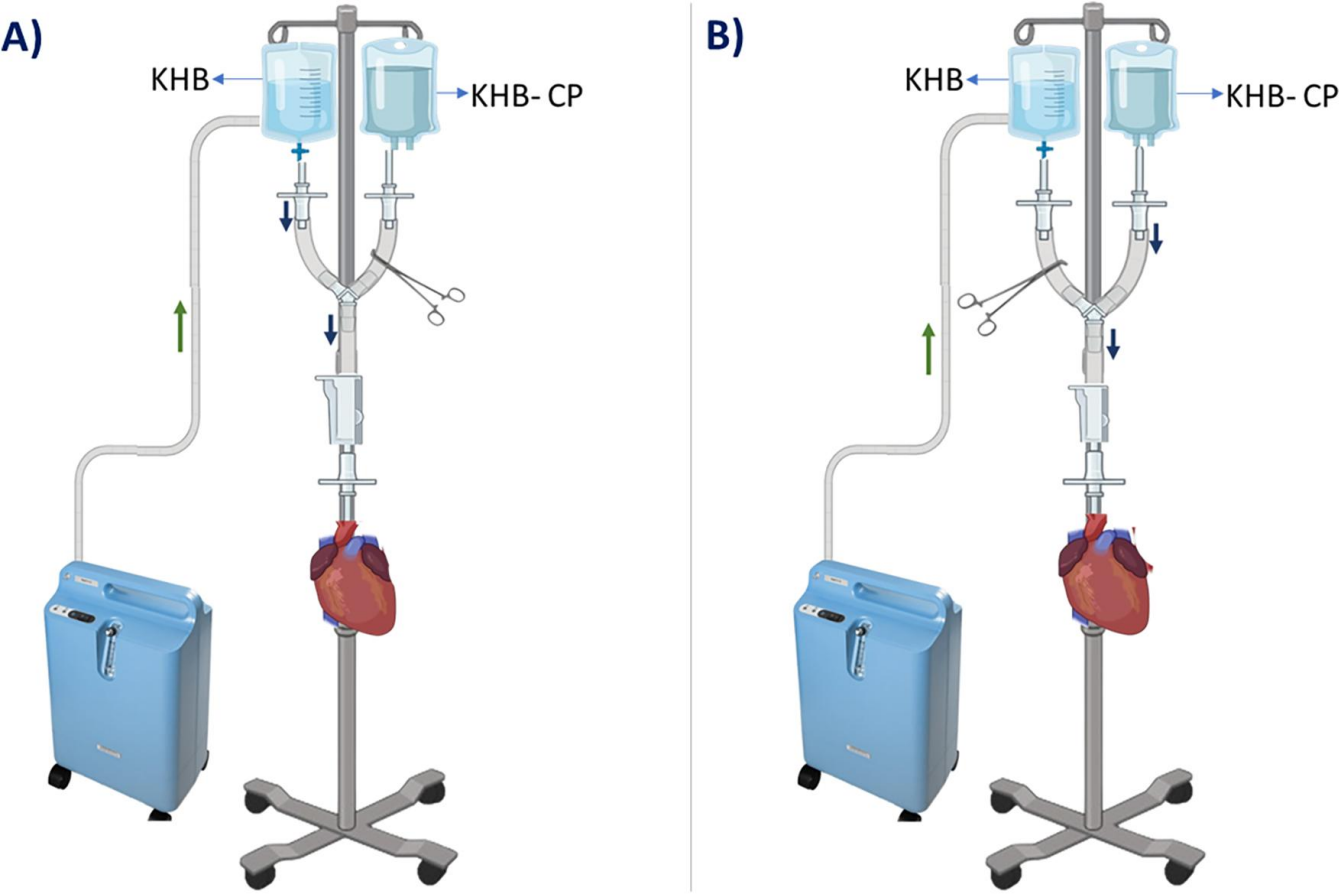
Representative images of the ex-vivo perfusion model in wistar rat hearts. **(A)** Heart during the stabilization/reperfusion phase, demonstrating functional recovery under perfusion conditions. **(B)** Induction of myocardial ischemic arrest using a Krebs–Henseleit buffer (KHB)-based cardioplegic solution.

Together, the two experimental groups allowed for rigorous evaluation of the I–R protocol, with the non-ischemic controls providing essential reference values for interpreting cardiac function and tissue viability following ischemia–reperfusion injury. (Figure 2).

### 2.6 Surgical procedure for isolation of rat heart with aorta

#### 2.6.1 Pre-dissection preparation

Rats were administered heparin (1,000 IU/kg) at least 30 min prior to dissection, followed by euthanasia through an overdose of anesthetic agents. The animal was placed in the supine position on the surgical table. All required instruments, including cotton for blood removal, Krebs–Henseleit buffer (KHB) with oxygen supply, scalpel, toothed and blunt forceps, Jorgenson scissors, and Iris scissors, were prepared in advance.

#### 2.6.2 Initial abdominal and thoracic access

**Figure 3:**
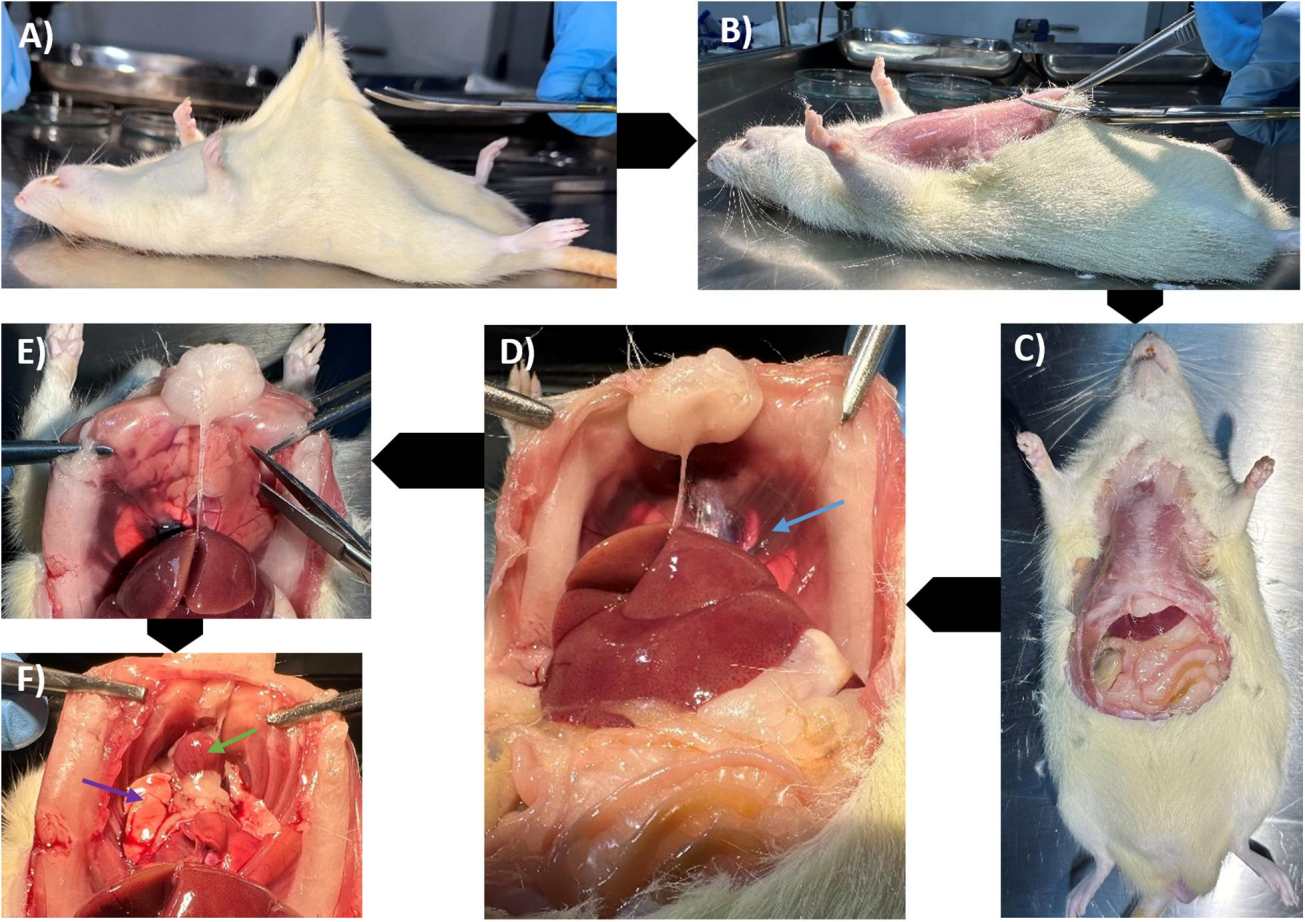
Sequential dissection of the rat to access abdominal and thoracic cavities. **(A)** Elevation of the upper abdominal skin with forceps prior to excision. **(B)** Removal of the anterior abdominal wall and overlying skin, extending cranially toward the neck. **(C)** Visualization of abdominal viscera following excision of the abdominal wall. **(D)** Inferior view showing the diaphragm (blue arrow) separating the abdominal and thoracic compartments. **(E)** Excision of the diaphragm to enter the thoracic cavity. **(F)** Inferior thoracic view after diaphragm removal, exposing the heart (green arrow) and lungs (purple arrow).

The skin of the upper abdomen was elevated using toothed forceps and excised with Jorgenson scissors, with the curve oriented away from the rat to prevent accidental injury to underlying organs (Figure 3A). The incision was extended cranially up to the neck, excising the anterior abdominal wall muscles together with the skin (Figure 3B). This exposed the inferior surface of the diaphragm (Figures 3C,D). The diaphragm margins were carefully excised with Iris scissors to access the thoracic cavity inferiorly (Figures 3E,F).

#### 2.6.3 Exposure of thoracic structures

**Figure 4:**
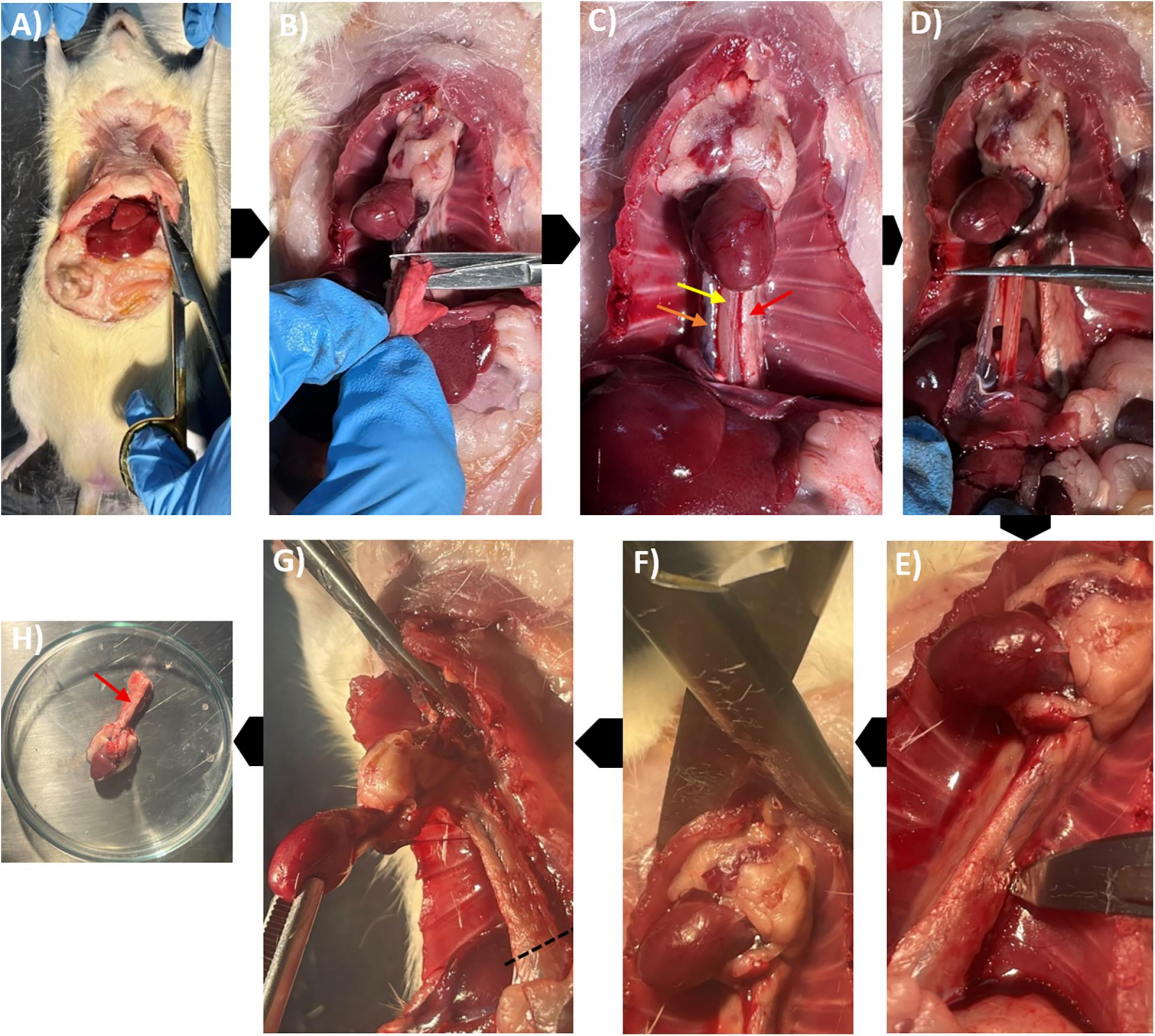
Sequential dissection of the thoracic cavity and isolation of the heart along with aorta. **(A)** Excision of the rib cage to expose thoracic organs. **(B)** Removal of the lungs to visualize mediastinal structures. **(C)** Thoracic cavity following lung removal showing the inferior vena cava (orange arrow), esophagus (yellow arrow), and aorta (red arrow). **(D)** Transection of the esophagus and inferior vena cava. **(E)** Dissection of the thoracic aorta from posterior vertebral attachments with a scalpel. **(F)** Transection of superior thoracic attachments, including the trachea, to free the aortic arch. **(G)** Final separation of posterior aortic attachments; dotted line indicates distal transection level for heart–aorta preparation. **(H)** Isolated heart with intact aorta (purple arrow) placed in Krebs–Henseleit buffer (KHB).

Using Jorgenson scissors with the curve directed outward, the rib cage was excised in a circular fashion to expose the thorax anteriorly (Figure 4A). The lungs were then retracted and removed using Iris scissors to visualize the posterior thoracic wall (Figure 4B). At this stage, three tubular structures were observed descending from the thorax to the abdomen: the inferior vena cava, the esophagus, and the aorta (Figure 4C). The aorta, firmly attached to the left side of the thoracic vertebra, was the primary structure of interest. To minimize confusion during mounting, the esophagus and inferior vena cava were transected close to the heart (Figure 4D), leaving only short remnants.

#### 2.6.4 Isolation and dissection of the heart with aorta

Following the exposure of thoracic structures, aorta was carefully dissected free from the vertebral attachments using a scalpel angled towards the vertebra to avoid vascular injury (Figure 4E). Dissection proceeded up to the aortic arch, avoiding traction on the vessel. Jorgenson scissors, oriented towards the rat's head, were then used to transect the upper thoracic attachments, including the trachea (Figure 4F). The heart was gently lifted with blunt forceps, and any remaining posterior connections of the thoracic aorta were severed with Iris scissors (Figure 4G). The aorta was then transected as distally as possible to retain sufficient length for mounting, and the isolated heart with the aorta was placed in a Petri dish containing KHB (Figure 4H).

The principle of this technique is to preserve a long segment of the aorta along with the heart while ensuring that all other tubular structures remain short to prevent misidentification. Distinguishing features of the aorta include its firm adherence to the vertebra via whitish-yellow connective tissue, its three major branches arising from the arch, and, in some cases, visible pulsation if cardiac activity persists. By contrast, the trachea can be identified by palpable cartilaginous rings, the esophagus appears bright red due to its muscular nature, and the inferior vena cava carries darker deoxygenated blood, courses close to the liver and terminates on the right side of the heart.

### 2.7 Mounting of the isolated heart on the *ex vivo* perfusion apparatus

**Figure 5:**
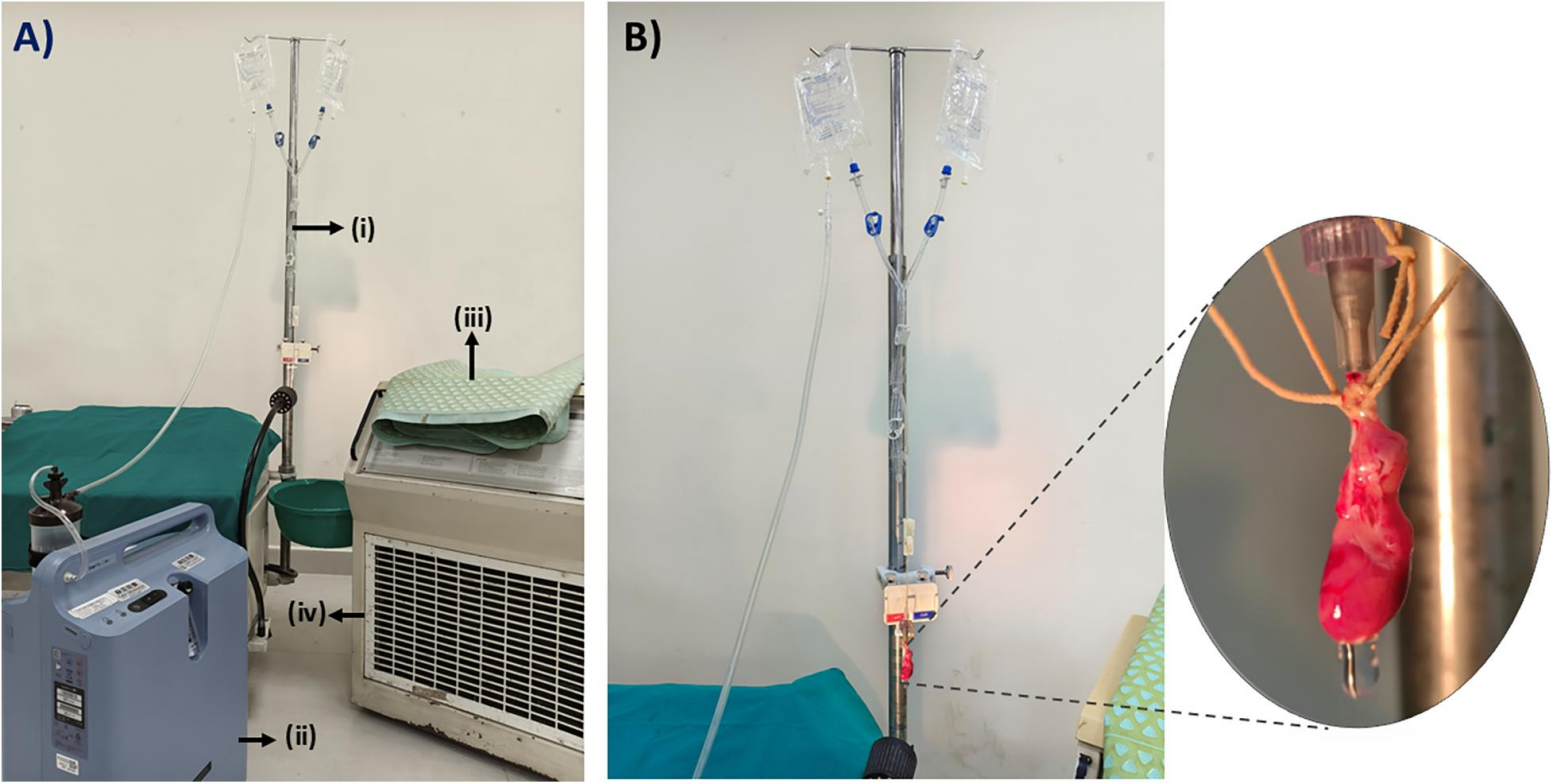
Images representing the complete Ex-vivo perfusion model. **(A)** Various components of ex-vivo perfusion model. **(i)** Extracorporeal circuit for ex-vivo perfusion model, **(ii)** An oxygen concentrator which provides a continuous flow of oxygenated perfusate, **(iii)** water blanket, **(iv)** Heater-cooler unit which regulates the water blanket temperature. **(B)** Mounted rat heart in the ex-vivo perfusion model.

Following careful trimming of extraneous tissues, the isolated heart along with aortic cuff was meticulously positioned and threaded onto a 20-gauge blunt cannula, which served as the aortic inflow conduit. The cannula was then securely fastened using a pre-placed suture to establish a leak-proof seal, ensuring uninterrupted perfusion. A brief retrograde flush of oxygenated KHB was performed to remove residual blood from the coronary vasculature and to prevent air embolism. During the entire mounting procedure, the myocardium was continuously bathed with oxygenated KHB to preserve tissue viability and minimize ischemic injury. The process, spanning from heart excision to successful cannulation and attachment to the *ex vivo* perfusion system, was completed within one minute, thereby limiting total warm ischemic time and preserving optimal functional integrity of the isolated heart (Figure 5).

### 2.8 Stabilization of the isolated heart

Following cannulation, heart of all the animals was then retrogradely perfused with KHB solution for 10 min as a stabilization period, with a constant perfusion pressure equivalent to 100 cm water (10 kPa) (28, 29). The perfusate was equilibrated with atmospheric air using an oxygen concentrator (Philips EverFlo) and maintained at 35 ± 2 °C throughout the procedure (9). The water blanket was supplied with circulating water maintained at 37 °C by the heater–cooler unit, and the KHB-filled bags were positioned within the rolled-up water blanket to facilitate efficient heat transfer from the circulating water in the water blanket to the perfusate (KHB solution). A thermometer was used to continuously monitor the perfusate temperature. Whenever a decline in perfusate temperature was noted, pre-warmed KHB-filled bags were replaced to ensure consistent thermal delivery, thereby maintaining the perfusate at 35 ± 2 °C throughout the entire ex-vivo procedure. Spontaneous beating was typically restored within 1–2 min of establishing perfusion.

### 2.9 Ischemic arrest and reperfusion

Following a 10 min stabilization period with normothermic KHB solution, isolated hearts of the I-R induced group were subjected to 30 min of global ischemic arrest. This included a 1 min delivery of normothermic KHB- CP solution to achieve diastolic arrest. During the KHB-CP delivery, the KHB perfusate was stopped by firmly applying a metal clamp. Upon completion of the arrest period, reperfusion was initiated with normothermic KHB solution for 60 min. During this reperfusion step, the metal clamp was removed from the KHB perfusate and applied to the KHB-CP perfusate. Throughout the entire procedure, KHB solution was sprayed onto the isolated heart to prevent drying of the myocardium.

### 2.10 Measurement of coronary flow rate

Coronary flow rate was assessed at two defined time points in both experimental groups to enable direct comparison. In the ischemia–reperfusion (I–R) group, coronary flow was measured first during the initial stabilization period, representing baseline conditions before ischemia, and again at the 5th minute of reperfusion phase following 30 min of global ischemic arrest. In the non-ischemic control group, coronary flow was recorded at the same two time points; however, these hearts remained under continuous physiological perfusion throughout the experiment without undergoing ischemia or reperfusion. This parallel measurement strategy allowed a clear distinction between normal coronary flow dynamics and the alterations induced by ischemia–reperfusion injury. Assessment was performed by timed collection of the coronary effluent exiting the aorta and dripping into a pre-weighed beaker. The effluent volume was accurately measured per minute using a calibrated pipette, and the coronary flow rate was expressed in mL/min (26). This method allowed real-time quantification of myocardial perfusion, providing insights into the functional recovery of the heart following ischemic insult and the efficacy of reperfusion. Continuous monitoring of coronary flow also served as an indirect indicator of vascular integrity and myocardial vi-ability during *ex vivo* perfusion.

### 2.11 Gross morphological assessment of isolated heart

Upon completion of the experimental protocol, the isolated hearts were gently rinsed with cold normal saline to remove residual blood and debris and examined under ambient lighting. The gross morphological assessment involved a detailed evaluation of heart size, shape, epicardial surface texture, and tissue coloration, which are indicative of overall myocardial integrity and perfusion status. Observations were systematically recorded to allow comparative analysis between control and ischemia-reperfusion groups, providing preliminary insight into potential structural alterations resulting from ischemic insult.

### 2.12 Myocardial infarct area assessment using 2,3,5-triphenyltetrazolium chloride (TTC) staining

Following gross examination, the hearts of all experimental animals were freshly sectioned transversely using a long, sharp blade at a standardized mid-ventricular level, identified by consistent internal anatomical landmarks (papillary muscle level). This approach minimized variability in slice position across animals. Infarct assessment was performed on these representative mid-ventricular sections to evaluate ischemic changes. The infarcted area was quantified on the same mid-ventricular slice in all animals and reported as a percentage of the total left-ventricular cross-sectional area.

Tissue slices were briefly rinsed under cold running water to remove surface clots and residual blood, taking care to minimize wash duration to preserve tissue morphology and prevent the elution of cellular contents. The slices were then transferred to glass jars sized to allow uniform exposure to the staining solution. A freshly prepared 2,3,5-triphenyltetrazolium chloride (TTC) solution was added to completely submerge the tissue, maintaining a fluid height approximately 2 cm above the slices (31). The slices were incubated in TTC at room temperature for 30 min in the dark, allowing for the enzymatic reduction of TTC by viable myocardial tissue, thereby differentiating viable myocardium from non-viable (infarct) areas. Post TTC staining, tissue fixation of mid ventricular sections was carried out in 10% formalin overnight, ensuring preservation of morphology and for further quantitative scoring to distinguish viable myocardium from infarcted (non-viable) tissue. The quantitative scoring was performed as follows: a score of 0 indicated no detectable infarction (0%); 1. corresponded to mild infarction involving 1%–25% of the myocardial area; 2. represented moderate infarction (26%–50%); 3. denoted extensive infarction (51%–75%); and 4. reflected severe or near-complete infarction involving 76%–100% of the examined tissue.

### 2.13 Histopathological analysis

Following 24 h of fixation in 10% formalin, mid-ventricular heart sections of all the experimental animals were subjected to graded ethanol dehydration: 50% ethanol for 48 h, 70% ethanol for 48 h, 90% ethanol for 24 h, and 100% ethanol for 24 h, ensuring complete removal of water and optimal tissue penetration. The tissues sections were subsequently cleared in xylene until transparency was achieved, followed by paraffin embedding using embedding rings. Tissue blocks were stored at −20 °C for 24 h to facilitate sectioning.

Histological sections of 5 *μ*m thickness were obtained using a rotary microtome and mounted on lysine-coated slides via a water bath to enhance tissue adherence. The mounted sections were dried on a hot plate and subjected to Hematoxylin and Eosin (H & E) staining, allowing detailed visualization of myocardial architecture, cellular integrity, and pathological alterations (32). All the slides were observed for the presence of degenerated cardiomyocytes, interstitial edema, cardiomyocyte cytoplasmic vacuolization, and mononuclear inflammatory cells infiltration. A validated scoring rubric (0–4) was applied to blinded slides. From each slide, 10 different fields were observed at ×400 magnification under the light microscope, and histopathological scoring was done as follows—absent (0), mild (1), moderate (2), and severe (3) (32). This comprehensive histopathological evaluation enabled correlation of macroscopic infarct characteristics with microscopic tissue changes, providing robust evidence for the impact of ischemia-reperfusion injury.

### 2.14 Statistical analysis

Statistical Package for the Social Sciences (SPSS) software was used to analyze all experimental data (Version 30.0). The quantitative data were presented in the form of mean ± standard deviation (SD). Continuous variables were analyzed using independent-samples *t*-test following assessment of normality. Semi-quantitative and ordinal data (e.g., TTC and histopathological scores) were analyzed using non-parametric tests (Mann–Whitney *U* test) to ensure appropriate statistical interpretation.

## 3 Results

### 3.1 Effect on coronary flow rate

Coronary flow assessment revealed clear distinctions in perfusion dynamics be-tween non-ischemic and ischemia–reperfusion (I–R) conditions. During the stabilization phase, the non-ischemic control and I–R groups exhibited comparable baseline coronary flow rates (12.25 ± 2.04 vs. 12.75 ± 0.94 mL/min, respectively), indicating that all hearts entered the experimental protocol under equivalent perfusion conditions. However, exposure to 30 min of global ischemia followed by reperfusion resulted in a profound impairment in coronary vascular function. At the 5th minute of reperfusion, the I–R group displayed a markedly reduced coronary flow of 2.70 ± 0.29 mL/min, rep-resenting a 4.7-fold significant decline (*p* < 0.001) relative to its own stabilization baseline and a 4.5-fold significant reduction (*p* < 0.001) when compared to NIC hearts at the corresponding time point (Table 2).

**Table 2:** Effect on coronary flow rate.

Experimental group	Physiological stage	Coronary flow (mL/min) (Mean ± SD)
Non-ischemic control	Stabilization	12.25 ± 2.04
I–R induced group	Stabilization	12.75 ± 0.94
I–R induced group	Reperfusion (5th minute)	2.70 ± 0.29***

***p ≤ 0.001 compared to the coronary flow rate during the stabilization in the I-R group.

### 3.2 Gross morphological examination of experimental hearts

**Figure 6:**
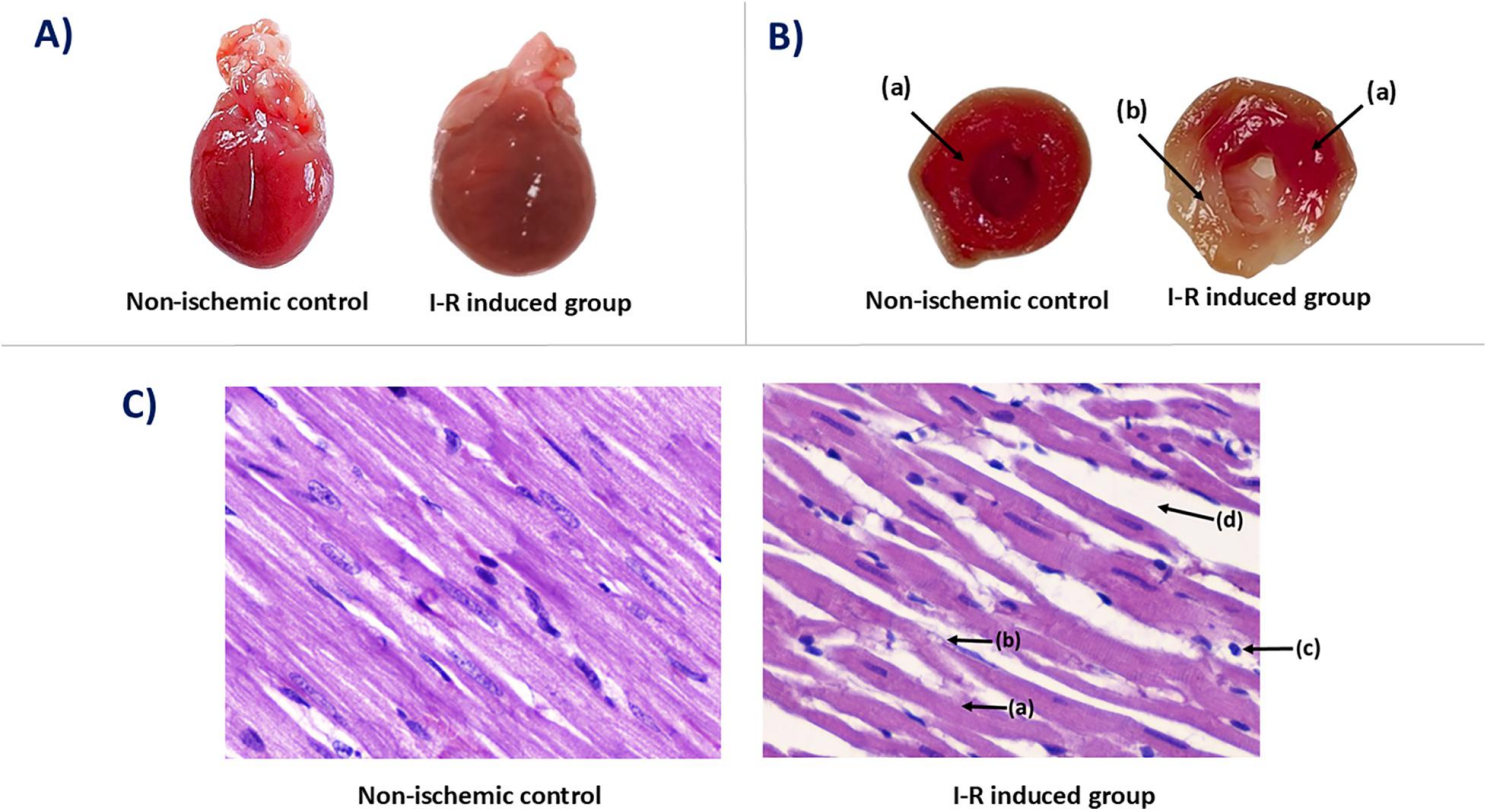
Gross morphology, TTC staining, and histopathological examination of Rat heart. **(A)** Gross morphological examination of cardiac tissues. **(B)** Assessment of the infarct area using TTC staining. The staining differentiates viable from non-viable tissue. **(a)** viable myocardium. **(b)** pale infarcted region. **(C)** Microscopic cellular architecture of cardiac tissue sections stained with Hematoxylin and Eosin (H&E). Images are from longitudinal sections at 400× magnification. Pathological features observed include **(a)** degenerated cardiomyocyte, **(b)** cardiomyocyte cytoplasmic vacuolization, **(c)** mononuclear inflammatory cell infiltration, and **(d)** interstitial edema.

Hearts from the non-ischemic control group exhibited normal size and contour, with a smooth and uniform epicardial surface. In contrast, hearts from the I-R induced group displayed mild enlargement and a mottled external appearance, characterized by irregular pale regions interspersed with dark congested areas, indicative of ischemia-reperfusion-induced tissue injury (Figure 6A).

### 3.3 Effect on cardiac tissue following TTC staining

TTC staining demonstrated a clear distinction in myocardial viability between the two groups when qualitative observations were interpreted alongside quantitative scoring. In the non-ischemic control group, myocardial tissue exhibited uniform, deep red staining, reflecting intact mitochondrial dehydrogenase activity and complete tissue viability. These qualitative findings were fully supported by the quantitative TTC score of 0.00 ± 0.00, confirming the absence of infarcted myocardium. In contrast, hearts subjected to ischemia–reperfusion (I–R) injury showed a heterogeneous staining pattern, with viable myocardium retaining the characteristic brick-red color while infarcted regions remained pale or unstained, indicating loss of enzymatic activity. This visual evidence corresponded with a significantly elevated infarct score of 2.20 ± 0.51 (*p* ≤ 0.001), consistent with extensive myocardial non-viability (approximately 55% involvement). Together, the qualitative and quantitative TTC results support the induction of myocardial injury within the experimental model (Figure 6B and Table 3).

**Table 3:** Quantitative scoring of TTC and H & E-stained myocardial tissues.

Groups	Scoring of TTC stained myocardial tissues (Mean ± SD)	Scoring of H & E-stained myocardial tissues (Mean ± SD)			
	Non-viable (infarct) myocardial tissue	Degenerated cardiomyocytes	Cardiomyocyte cytoplasmic vacuolization	Mononuclear inflammatory cells infiltration	Interstitial edema
Non-ischemic control	0.00 ± 0.00	0.00 ± 0.00	0.00 ± 0.00	0.16 ± 0.40	0.16 ± 0.40
IR- induced group	2.20 ± 0.51***	2.16 ± 0.40***	2.00 ± 0.00***	1.66 ± 1.21*	2.66 ± 0.51***

***p ≤ 0.001, and *p ≤ 0.05.

### 3.4 Histopathological findings of myocardial sections

Histopathological examination using H&E staining revealed preserved myocardial structure in the non-ischemic control group, characterized by intact muscle fibers, clear cross-striations, centrally located nuclei, and absence of structural abnormalities. These qualitative observations aligned with the near-zero quantitative scores for cardiomyocyte degeneration (0.00 ± 0.00), cytoplasmic vacuolization (0.00 ± 0.00), and interstitial edema (0.16 ± 0.40). Only minimal inflammatory cell infiltration was observed (0.16 ± 0.40), consistent with normal myocardial morphology. In parallel, myocardial sections from the I–R group demonstrated pronounced pathological alterations including multifocal cardiomyocyte degeneration, hypereosinophilic cytoplasm, loss of nuclear detail, and prominent cytoplasmic vacuolization. The interstitial spaces revealed marked edema accompanied by mononuclear inflammatory cell infiltration. These qualitative abnormalities corresponded closely with their respective quantitative injury scores: cardiomyocyte degeneration (2.16 ± 0.40; *p* ≤ 0.001), cytoplasmic vacuolization (2.00 ± 0.00; *p* ≤ 0.001), mononuclear inflammatory cells infiltration (1.66 ± 1.21; *p* ≤ 0.05), and interstitial edema (2.66 ± 0.51; *p* ≤ 0.001). The histopathological changes shown in Figure 6C are representative of the injury pattern within the affected myocardium, rather than isolated focal abnormalities. The strong agreement between microscopic observations and quantitative grading confirms significant structural damage induced by ischemia–reperfusion (Figure 6C and Table 3).

## 4 Discussion

The present study establishes a resource-conscious proof-of-concept *ex vivo* perfusion model capable of reproducing key features of myocardial ischemia–reperfusion injury. This model effectively replicates the key physiological and pathophysiological hallmarks of myocardial IRI, including compromised coronary perfusion, infarct formation, and cellular degeneration (33, 34), while utilizing cost-effective, readily available components. The novelty of this approach lies not only in its technical simplicity and resource-conscious design but also in its ability to demonstrate consistent experimental performance within controlled conditions; however, broader reproducibility and translational applicability require further validation. By bridging the gap between high-resource laboratories and those with infrastructural limitations, the study offers an accessible platform for preclinical cardiovascular research, facilitating mechanistic exploration and evaluation of therapeutic strategies.

The Langendorff ex-vivo perfusion system has long served as a cornerstone in cardiovascular research (35, 36), allowing for the isolation of intrinsic cardiac function from systemic influences, including neurohormonal modulation, circulating metabolites, and inter-organ interactions. While conventional setups provide high precision, their cost and technical complexity limit widespread adoption, particularly in resource-constrained environments. The present model introduces methodological refinements that enhance reproducibility and minimize variability, including rapid heart excision, precise cannulation, controlled mounting on the perfusion system, continuous myocardial hydration with oxygenated Krebs-Henseleit buffer (KHB), and strict adherence to standardized ischemia and reperfusion protocols. These procedural improvements collectively ensure that the isolated heart retains contractile activity, coronary perfusion, and tissue viability, thereby maintaining essential functional characteristics, although not fully comparable to advanced Langendorff systems or *in vivo* physiology. The study's novelty also extends to the demonstration that these refinements, when implemented with readily available laboratory materials, can replicate the core pathophysiological features of IRI, making advanced cardiovascular experimentation more feasible across diverse research settings.

The key objective of the present study was to address the economic and infrastructural barriers associated with conventional *ex vivo* cardiac perfusion systems. To substantiate this aspect, a comparative analysis of cost, infrastructure, and operational requirements between the proposed simplified model and the conventional Langendorff system is presented (Table 4). The simplified system demonstrates a substantial reduction in capital investment, maintenance burden, and technical complexity, thereby enhancing accessibility for laboratories with limited resources (4–8, 35, 36).

**Table 4:** Comprehensive cost, infrastructure, and operational comparison between the proposed resource-conscious *ex vivo* perfusion model and conventional Langendorff system.

Category	Parameter	Proposed simplified *ex vivo* model	Conventional langendorff system	Scientific/operational implication
Capital cost	Initial setup cost	Very low (USD 30–50; reusable basic lab materials)	High (USD 32,962–95,302 depending on system)	Major barrier reduction for low-resource labs
	Specialized equipment	Not required	Essential (pressure transducers, perfusion pumps, data acquisition systems)	Limits advanced measurements in simplified model
	Procurement complexity	Low (locally available components)	High (imported specialized systems)	Faster implementation
Operational cost	Per experiment cost	Low	Moderate to high	Enables high-throughput screening
	Maintenance cost	Minimal/negligible	High (calibration, servicing)	Long-term sustainability advantage
	Consumables	Standard lab consumables	Specialized tubing, sensors, reagents	Cost variability higher in Langendorff
Infrastructure requirements	Lab setup	Basic laboratory environment sufficient	Advanced lab with controlled perfusion environment required	Accessibility advantage
	Power & backup systems	Minimal dependence	Continuous power supply essential	Operational risk lower in simplified model
	Space requirements	Compact setup	Dedicated setup area required	Space efficiency
Technical expertise	Skill requirement	Moderate (trainable within short duration)	High (specialized training required)	Lower learning curve
	Setup time	Short (minutes)	Long (system calibration required)	Efficiency advantage
	Reproducibility control	Operator-dependent	System-controlled high precision	Potential variability in simplified model
Physiological measurements	Coronary flow	Yes (manual measurement)	Yes (automated, high precision)	Basic vs. advanced accuracy
	Hemodynamic parameters (LV pressure, dP/dt)	Not available	Available	Major limitation of simplified model
	ECG monitoring	Not available	Available	Limits electrophysiological studies
Experimental capability	Model type	Proof-of-concept injury induction	Full physiological modeling	Defines scope of use
	Mechanistic studies	Limited	Extensive	Impacts publication depth
	Drug testing	Preliminary screening	Advanced pharmacological evaluation	Screening vs. validation
Scalability	Small animals (rats/mice)	Highly feasible	Standard use	Comparable
	Large animals (rabbits, pigs)	Not feasible without major modification	Feasible	Key limitation
	Human heart application	Not feasible	Feasible (research settings)	Translational limitation
Data quality & precision	Measurement precision	Moderate	High	Impacts reproducibility
	Automation	Absent	High automation	Operator bias in simplified model
Ethical & training utility	Training tool	Highly suitable for beginners	Requires prior expertise	Educational advantage
	Ethical compliance burden	Standard	Standard	No major difference
Overall utility	Accessibility	High	Limited	Major advantage
	Translational relevance	Preliminary	High	Must be clearly stated
	Research stage suitability	Early-stage/proof-of-concept	Advanced/validation studies	Defines positioning

While the proposed model offers clear advantages in affordability and ease of implementation, it is important to acknowledge that these benefits come at the cost of reduced physiological precision and limited functional measurements. Unlike conventional Langendorff systems, which enable detailed hemodynamic and electrophysiological assessments, the present model is primarily suited for proof-of-concept studies and preliminary screening of cardioprotective interventions. Therefore, it should be viewed as a complementary approach rather than a replacement for established perfusion systems (35, 36).

Functional assessment of coronary flow rate provides an essential metric for evaluating myocardial perfusion and vascular integrity (28, 31). In the present study, a marked reduction in coronary flow rate was observed during reperfusion in the I-R group compared to the initial stabilization period. This decrease reflects the impact of ischemic insult on coronary vasculature and hemodynamic recovery, consistent with phenomena such as endothelial dysfunction, microvascular obstruction, and capillary collapse, which have been reported in both experimental and clinical contexts of reperfusion injury (28, 37, 38). The use of timed effluent collection to quantify flow rate in mL/min allowed for sensitive and reproducible assessment of functional impairment. This functional readout, when integrated with morphological and histological evaluations, provides a multidimensional understanding of myocardial injury and validates the model as a reliable platform for testing cardioprotective interventions.

Gross morphological examination revealed distinct differences between non-ischemic controls and I-R hearts. Control hearts exhibited normal size, contour, and smooth epicardial surfaces, reflecting preserved structural integrity. In contrast, I-R hearts displayed mild enlargement, uneven epicardial texture, and patchy discoloration with pale and congested regions, indicative of ischemia-reperfusion-induced tissue injury (Figure 6A). These macroscopic alterations, although informative, may not fully capture the extent of myocardial damage at the cellular or molecular level, underscoring the importance of complementary assessments. Nonetheless, the reproducibility of these morphological changes across hearts in the I-R group emphasizes the robustness of the standardized experimental procedure.

Assessment of myocardial viability using 2,3,5-triphenyltetrazolium chloride (TTC) staining provided precise delineation between viable and non-viable tissue (39). TTC is reduced by mitochondrial dehydrogenases in metabolically active cells, forming a brick-red formazan precipitate, while infarcted regions lacking enzymatic activity remain pale (40, 41). In this study, non-ischemic hearts exhibited uniform bright-red staining, confirming intact mitochondrial function and myocardial viability. Conversely, I-R hearts showed heterogeneous staining patterns, with brick-red viable myocardium interspersed with pale infarcted regions, validating the reproducibility and physiological relevance of the model. The integration of functional assessment with TTC staining enables a comprehensive evaluation of myocardial injury and provides a reliable platform for the preclinical testing of cardioprotective agents (42, 43). The novelty of this approach lies in demonstrating that such precise viability assessments can be achieved using a cost-effective and simplified ex-vivo perfusion setup.

Histopathological evaluation using Hematoxylin and Eosin staining further elucidated cellular-level changes associated with IRI. Non-ischemic hearts retained normal architecture, with well-organized myofibers, intact cross-striations, and centrally located nuclei. In contrast, I-R hearts exhibited multifocal cardiomyocyte degeneration characterized by hyper-eosinophilic cytoplasm, nuclear pyknosis, and cytoplasmic vacuolization. Interstitial edema and focal mononuclear inflammatory infiltration were evident, reflecting reperfusion-mediated injury, oxidative stress, and inflammatory activation (22, 23, 44–49). The congruence between TTC-delineated infarcts and histological findings strengthens confidence in the model's reliability, highlighting its capacity to reproduce both structural and cellular manifestations of myocardial ischemia-reperfusion injury. These results demonstrate the strength of the model in capturing the spectrum of myocardial alterations, from macrostructural features to microscopic cellular pathology, thereby providing a comprehensive framework for mechanistic studies.

Ex-vivo myocardial perfusion models remain a cornerstone of preclinical cardioprotection research; however, traditional Langendorff systems are prohibitively expensive for many laboratories, particularly in low-resource regions. By designing a fully functional, reproducible, physiologically valid, and substantially more affordable alternative, our study directly addresses a global accessibility barrier. This model enables wider participation in preclinical ischemia–reperfusion research, facilitates training in surgical and perfusion techniques, and supports early-phase testing of candidate cardioprotective compounds. The translational impact lies in democratizing access to experimental cardiology platforms, thereby accelerating scientific contribution from institutions that currently face technological and financial limitations.

This ex-vivo perfusion platform is inherently scalable and can be adapted to a wide range of species and disease phenotypes with minor technical modifications. For smaller rodents such as mice, scaling considerations include the use of a finer aortic cannula, lower perfusion pressures (typically 60–70 mmHg), and reduced absolute coronary flow to match species-specific hemodynamic requirements. Conversely, for rabbits and larger rodents, a proportionally larger cannula and higher flow capacities can be employed to maintain physiological perfusion. The scalability of this model to larger animals such as pigs or sheep is limited due to the need for precise pressure control, oxygenation systems, and advanced perfusion circuitry. Therefore, direct extrapolation to large-animal or human heart models is not feasible without substantial modification. The model can also be tailored to pathological conditions: in diabetic myocardium, the Krebs–Henseleit buffer may be supplemented with elevated glucose concentrations and advanced glycation end-products to mimic the metabolic milieu characteristic of chronic hyperglycemia; in hypertensive hearts, longer stabilization periods and controlled augmentation of afterload can be incorporated to simulate the altered ventricular mechanics associated with sustained pressure overload. These adjustments collectively demonstrate the flexibility of the platform and its suitability for diverse mechanistic and translational cardiovascular research applications.

One of the major strengths of the present study is the integration of multiple assessment modalities; functional, gross morphological, TTC staining, and histopathology to provide a holistic evaluation of myocardial injury. This multi-tiered approach enhances interpretive rigor, allowing correlation between functional deficits, infarct formation, and cellular damage. Additionally, the standardized procedural protocol minimizes inter-animal variability, increasing the reproducibility and reliability of outcomes. The model's cost-effective design represents another key strength, making it accessible for laboratories with limited resources without compromising scientific integrity.

Nevertheless, the study has certain limitations. First, it represents a proof-of-concept model with a small sample size, limiting generalizability. Second, advanced functional parameters such as ventricular pressure measurements were not assessed. Third, the model does not replicate the precision of conventional Langendorff systems. Fourth, semi-quantitative scoring methods introduce inherent variability, although appropriate statistical corrections were applied. Finally, translational extrapolation should be made cautiously.

Despite these limitations, the study demonstrates the novelty, robustness, and translational relevance of the proposed ex-vivo perfusion model. By faithfully replicating functional, macroscopic, and cellular hallmarks of myocardial IRI, the model enables mechanistic investigations and preclinical therapeutic evaluations with high reproducibility. Its simplicity and cost-effectiveness expand accessibility, enabling laboratories across diverse settings to perform high-quality cardiovascular research, thus fostering collaborative investigations and accelerating translational discovery. Furthermore, the procedural refinements introduced herein, such as rapid heart excision, precise aortic cannulation, continuous myocardial hydration, and standardized ischemia-reperfusion timing, represent methodological innovations that can be adopted in other resource-conscious experimental designs.

## 5 Conclusion

The present study provides a validated, reliable, and accessible ex-vivo perfusion model of myocardial ischemia-reperfusion injury, bridging the gap between high-resource and resource-limited research environments. The model reproduces the core functional, structural, and cellular features of IRI while remaining practical and cost-effective. Its reproducibility, combined with integrated functional, gross morphological, viability, and histopathological assessments, ensures a comprehensive evaluation of myocardial injury and supports mechanistic exploration and therapeutic testing. By emphasizing both scientific rigor and accessibility, this model has the potential to broaden global research capacity, facilitate translational cardiovascular investigations, and accelerate the development of effective strategies to mitigate ischemia-reperfusion injury in clinical settings.

## Data Availability

The original contributions presented in the study are included in the article/Supplementary Material, further inquiries can be directed to the corresponding author.
